# Using Standardized Interpretation of Chest Radiographs to Identify Adults with Bacterial Pneumonia—Guatemala, 2007–2012

**DOI:** 10.1371/journal.pone.0133257

**Published:** 2015-07-24

**Authors:** Jonathan M. Wortham, Jennifer Gray, Jennifer Verani, Carmen Lucia Contreras, Chris Bernart, Fabiola Moscoso, Juan Carlos Moir, Emma Lissette Reyes Marroquin, Rigoberto Castellan, Wences Arvelo, Kim Lindblade, John P. McCracken

**Affiliations:** 1 Centers for Disease Control and Prevention, Atlanta, Georgia, United States of America; 2 Universidad del Valle de Guatemala, Guatemala City, Guatemala; 3 Ministerio de Salud Pública y Asistencia Social, Guatemala City, Guatemala; Weill Medical College of Cornell University, UNITED STATES

## Abstract

**Background:**

Bacterial pneumonia is a leading cause of illness and death worldwide, but quantifying its burden is difficult due to insensitive diagnostics. Although World Health Organization (WHO) protocol standardizes pediatric chest radiograph (CXR) interpretation for epidemiologic studies of bacterial pneumonia, its validity in adults is unknown.

**Methods:**

Patients (age ≥15 years) admitted with respiratory infections to two Guatemalan hospitals between November 2007 and March 2012 had urine and nasopharyngeal/oropharyngeal (NP/OP) swabs collected; blood cultures and CXR were also performed at physician clinical discretion. ‘Any bacterial infection’ was defined as a positive urine pneumococcal antigen test, isolation of a bacterial pneumonia pathogen from blood culture, or detection of an atypical bacterial pathogen by polymerase chain reaction (PCR) of nasopharyngeal/oropharyngeal (NP/OP) specimens. ‘Viral infection’ was defined as detection of viral pathogens by PCR of NP/OP specimens. CXRs were interpreted according to the WHO protocol as having ‘endpoint consolidation’, ‘other infiltrate’, or ‘normal’ findings. We examined associations between bacterial and viral infections and endpoint consolidation.

**Findings:**

Urine antigen and/or blood culture results were available for 721 patients with CXR interpretations; of these, 385 (53%) had endpoint consolidation and 253 (35%) had other infiltrate. Any bacterial infection was detected in 119 (17%) patients, including 106 (89%) pneumococcal infections. Any bacterial infection (Diagnostic Odds Ratio [DOR] = 2.9; 95% confidence Interval (CI): 1.3–7.9) and pneumococcal infection (DOR = 3.4; 95% CI: 1.5–10.0) were associated with ‘endpoint consolidation’, but not ‘other infiltrate’ (DOR = 1.7; 95% CI: 0.7–4.9, and 1.7; 95% CI: 0.7–4.9 respectively). Viral infection was not significantly associated with ‘endpoint consolidation’, ‘other infiltrate,’ or ‘normal’ findings.

**Interpretation:**

‘Endpoint consolidation’ was associated with ‘any bacterial infection,’ specifically pneumococcal infection. Therefore, endpoint consolidation may be a useful surrogate for studies measuring the impact of interventions, such as conjugate vaccines, against bacterial pneumonia.

## Introduction

Bacterial pneumonia is an important cause of morbidity and mortality worldwide and *Streptococcus pneumoniae* is the most common etiology, accounting for more than 30% of cases where an etiology was determined [1, 2]. The use of the pneumococcal conjugate vaccine in routine infant immunization programs has led to a decrease in invasive pneumococcal disease among unvaccinated adults in developed countries [3–6]. It is not known whether similar indirect (or herd) protection will be observed in low- and middle-income countries where the vaccine has been introduced [7], and where the burden of pneumococcal disease is much higher [8]. The magnitude of indirect effects from pneumococcal conjugate vaccine may greatly impact its cost-effectiveness and decisions about vaccine policy.

Accurately measuring the burden of bacterial, and specifically pneumococcal, pneumonia is challenging [9, 10]. While definitions based on isolation of bacterial pathogens from blood culture are very specific, they are insensitive because only a small fraction of patients with bacterial pneumonia have concurrent bacteremia [11–15]. Prior antimicrobial use, suboptimal sample collection, improper specimen handling, and inadequate laboratory conditions for isolation of fastidious organisms further limit the sensitivity of blood culture for detecting bacterial pneumonia, particularly in resource-limited settings. Definitions based on clinical signs and symptoms are sensitive for bacterial pneumonia, but they lack specificity, as viral pneumonia and other clinical syndromes cannot be distinguished from bacterial pneumonia [10, 16]. Sputum culture offers another option for the diagnosis of bacterial pneumonia, but adequate specimens are only obtained in a small proportion of cases, limiting its use as a tool to measure pneumonia burden [17]. While chest radiographs (CXR) are widely used for diagnosis of pneumonia, variability in interpretation limits the validity of radiologic findings as epidemiologic endpoints in studies of bacterial pneumonia [18].

The World Health Organization Department of Immunization, Vaccines, and Biologicals developed a standardized approach for interpretation of CXRs for epidemiologic studies of pneumonia in children and evaluations of vaccines against pneumonia [19–20]. The protocol describes specific radiographic findings considered to be indicative of bacterial pneumonia in children based on studies correlating these findings with bacterial infection [21–23]. Standardized interpretation is performed by a panel of CXR readers trained in the standardized approach; each reader independently reviews the CXR without taking into consideration clinical information about patients. CXRs are classified into three diagnostic categories: ‘endpoint consolidation’, meaning that the CXR has findings consistent with a bacterial etiology; ‘other infiltrate’, meaning that the CXR has abnormalities, but does not meet the criteria for endpoint consolidation; and ‘no consolidation/infiltrate/effusion’, indicating the absence of these radiologic findings. Standardized interpretation has been used as an epidemiologic tool to measure the efficacy of pneumococcal conjugate vaccines as well as impact and effectiveness of *Haemophilus influenzae* type B conjugate vaccines in clinical trials in young children [24–25]. This approach could also be helpful in the assessment of non-vaccine interventions for bacterial pneumonia.

There is limited experience with standardized interpretation of adult CXRs [26]. Adults have a higher prevalence of chronic medical conditions, such as congestive heart failure, that can cause radiographic findings similar to those found with bacterial pneumonia. The pathogens causing pneumonia in adults also more commonly include “atypical” bacteria such as *Mycoplasma pneumoniae* that may cause lobar consolidation, more classically associated with bacterial pneumonia, or an interstitial infiltrate, more classically associated with viral pneumonia [2, 27]. An evaluation of the WHO standardized interpretation is needed to determine whether this approach provides a useful tool for detection of bacterial pneumonia in adults. We sought to characterize the performance of standardized interpretation for an epidemiologic endpoint for bacterial pneumonia.

## Methods

We used data from a hospital-based respiratory disease surveillance system in Guatemala to examine associations between WHO CXR classifications and etiologies of respiratory infection among patients aged ≥15 years. The surveillance system, which has been described in previous work documenting etiologies of respiratory illnesses, [28] has two sites: Santa Rosa, which is located in the south of the country, and Quetzaltenango, in the western highlands. Surveillance is conducted at the primary public hospital in each of the departments and both facilities serve as regional reference hospitals. At the hospitals, study nurses reviewed ward registers for patients admitted for respiratory-related diagnoses as well as emergency department logs for patients presenting with respiratory complaints. After study nurses obtain verbal consent, they screen patients admitted with a respiratory-related admission diagnosis or chief complaint for inclusion as acute respiratory infection cases. Cases of hospitalized acute respiratory infections are defined as evidence of acute infection and signs or symptoms of respiratory disease (Table 1) occurring among patients of all ages admitted to one of the surveillance hospitals. After enrollment, demographic and clinical information are obtained from chart review and through patient interviews. During 2007–February 2012, study physicians performed a respiratory physical examination on all patients who met the case definition and documented pertinent respiratory system findings, such as rales and wheezes, using a standardized data collection form. Starting in March 2012, physical examinations are no longer performed by study physicians, but trained study nurses abstract pertinent respiratory findings on physical examination from the patient medical record and document whether or not patients died during their hospitalization. Nasopharyngeal and oropharyngeal (NP/OP) swabs are collected by trained study nurses according to CDC protocols from patients of all ages [27]; these swabs are tested for adenovirus, parainfluenza virus 1/2/3, respiratory syncytial virus, influenza A/B, human metapnuemovirus, *Chlamydophila pneumoniae*, and *Mycoplasma pneumoniae* by polymerase chain reaction (PCR). Urine specimens are collected only from patients ≥15 years old and are tested for *S*. *pneumoniae* using the Binax NOW (Alere, Traverse City, Michigan) urine antigen assay [28].

**Table 1:** Acute Respiratory Infection Case Definition*.

Evidence of acute infection	Signs or symptoms of respiratory disease
Fever (≥38°C)	Tachypnea
White blood cell (WBC) count < 3000 or >11000	Cough
Abnormal WBC differential	Sputum production
	Pleuritic chest pain
	Hemoptysis
	Difficulty breathing
	Shortness of breath
	Sore throat

* Hospitalized patients were considered to have acute respiratory disease if they met one or more of the criteria for “evidence of acute infection” AND one or more of the criteria for “signs or symptoms of respiratory disease.”

Blood cultures and CXRs are ordered at the discretion of the treating physician; CXR images are captured using a digital camera and are sent to [29] two radiologists who independently conduct a standardized interpretation of each image. CXRs with dense, lobar consolidation and/or a pleural effusion either on the same side as a pulmonary infiltrate or large enough to obscure opacity, if one were present, are categorized as ‘endpoint consolidation’. Those with a pulmonary infiltrate or effusion that did not meet the criteria for endpoint consolidation are categorized as ‘other infiltrate’. CXRs without findings consistent with ‘endpoint consolidation’ or ‘other infiltrate’ are categorized as ‘no consolidation/infiltrate/effusion’, or ‘normal’. CXRs whose features do not allow the interpretation of the endpoint consolidation characteristics are considered uninterpretable, according to the WHO protocol [20]. A third radiologist interprets CXRs for which the results of the first two interpretations are discordant, as well as 10% of images with concordant interpretations for quality control. All three radiologists were trained in the WHO standardized interpretation methodology. For this analysis, a final conclusion was derived for each CXR based on radiologists’ individual interpretations. A conclusion of ‘endpoint consolidation’ required the agreement of at least two radiologists; a conclusion of endpoint consolidation from only one of the three radiologists was classified as ‘other infiltrate’. CXRs were interpreted as ‘normal’ if all radiologists agreed that there were no findings consistent with ‘endpoint consolidation’ or ‘other infiltrate’.

Hospitalized patients with acute respiratory infections were considered to have ‘pneumococcal infection’ if their urine antigen test was positive or if *S*. *pneumoniae* was isolated from blood culture. Patients were considered to have a ‘typical bacterial infection’ if they had either evidence of a pneumococcal infection or if their blood culture grew any of the following pathogens: *Haemophilus influenzae*, *Moraxella cattharalis*, *Staphylococcus aureus*, *Pseudomonas aeruginosa*, and *Klebsiella pneumoniae*. Patients with a ‘typical bacterial infection’ or detection of *M*. *pneumoniae or C*. *pneumoniae* on NP/OP swabs were classified as having ‘any bacterial infection.’ Patients who did not meet definitions for ‘pneumococcal infection’, ‘typical bacterial infection’, or ‘any bacterial infection’, but who had a positive PCR for a viral pathogen were considered to have ‘viral infection.’

The surveillance protocol received approval from the institutional review boards of Universidad del Valle de Guatemala (Guatemala City, Guatemala), Centers for Disease Control and Prevention (CDC) (Atlanta, GA, USA), and the Guatemala Ministry of Public Health and Welfare. Verbal consent was requested from patients when screening them for eligibility. Written, informed consent was obtained from eligible patients willing to participate. For patients <18 years of age, parents or guardians were asked to provide written, informed consent for the participation of the patient, and children aged seven through 17 years were asked for written, informed assent.

In our analysis, we included only cases of hospitalized acute respiratory infection among adult patients aged ≥15 years with a standardized CXR interpretation and blood culture and/or *S*. *pneumoniae* urine antigen results available. We described demographic and clinical characteristics of patients, stratified by whether they had CXR with endpoint consolidation, other infiltrate, or normal patterns. We examined associations between the radiographic classifications (‘endpoint consolidation’, ‘other infiltrate’, or ‘normal’) and etiologies (‘pneumococcal infection’, ‘typical bacterial infection’, ‘any bacterial infection‘, and ‘viral infection’) using multivariable logistic regression with etiology as the dependent variable to calculate a diagnostic odds ratio [30]. To adjust for potential confounders of associations between radiographic pattern and etiology, we developed multivariable models, assessing patient characteristic variables associated with endpoint consolidation with p<0.10 in unadjusted models for possible inclusion in the adjusted models. We also quantified the sensitivity and specificity of endpoint consolidation for detecting ‘pneumococcal infection’, ‘typical bacterial infection’, and ‘any bacterial infection’. We excluded patients with uninterpretable chest radiographs from the analysis of sensitivity and specificity. All analyses were performed in SAS 9.3 (SAS Institute, Cary, NC).

## Results

**Fig 1:**
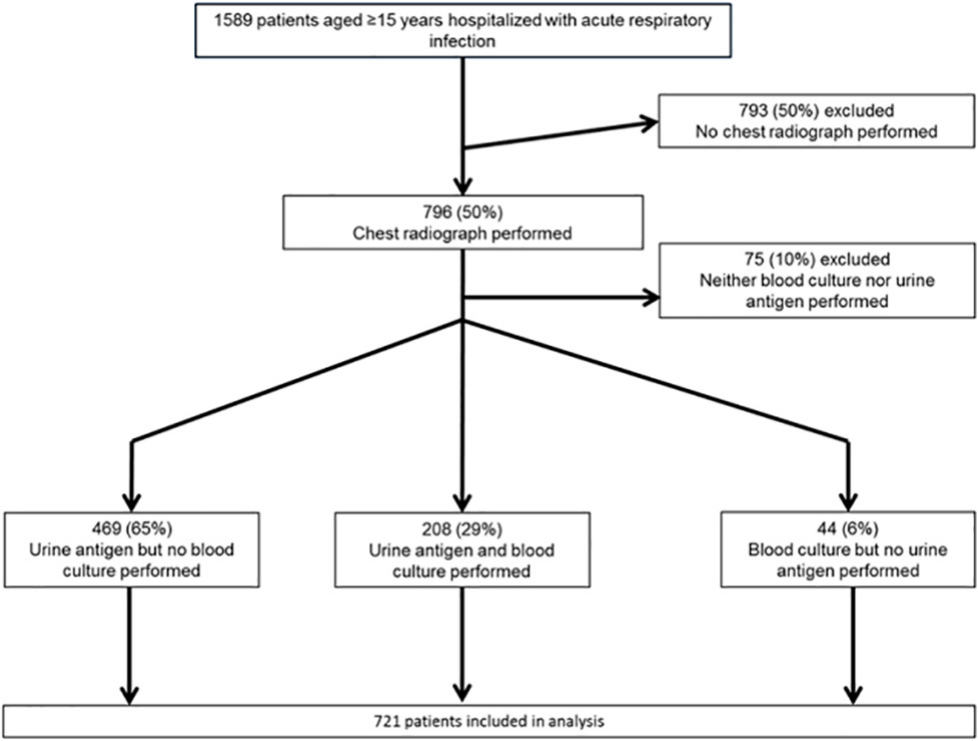
Patients included in the Analysis of Performance Characteristics of Standardized Interpretation. Of 1589 patients aged ≥15 years who were hospitalized with acute respiratory infection, 793 (50%) were excluded because no chest radiograph with which to perform standardized interpretation was performed. Of the remaining 796, 75 (10%) were excluded because no blood culture or urine antigen results were available. This left 721 patients who were included in the analysis; of these 469 (65%) had urine antigen results but no blood culture results, 208 (29%) had urine antigen and blood culture results, and 44 (6%) had blood culture results without urine antigen results.

Between November 2007 and March 2012, 1,589 consenting patients aged ≥15 years hospitalized with acute respiratory infection were enrolled in the surveillance system. CXRs were available for 796 (50%) patients. While a higher proportion of those who had a chest radiograph had rales (p<0.01), there were no significant differences between patients who had CXR and those who did not with respect to age (p = 0.75), sex (p = 0.43), ethnicity (p = 0.53), smoking status (p = 0.29), chronic conditions (p = 0.56), fever (p = 0.10), proportion that needed ICU admission (p = 0.09), or proportion that died (p = 0.68). Of the 796 patients with CXR, 721 (91%) had either blood culture or urine antigen testing and were included in the analysis (Fig 1). Among 721 patients included in the analysis, 345 (48%) were male, the median age was 54 years (range: 15–96 years), and 713 (99%) had testing of NP/OP specimens for viral and atypical bacterial pathogens performed.

Overall, 385 (53%) patients had endpoint consolidation on standardized interpretation, 253 (35%) had other infiltrate, and 79 (11%) were normal. Four CXRs (<1%) were uninterpretable. Demographic and clinical variables are presented in Table 2. Compared with having a normal CXR, having endpoint consolidation was associated with a higher median age (p<0.01), male sex (p<0.01), and history of smoking (p<0.04). There was no statistically significant difference between the proportion of patients with endpoint consolidation and those with normal CXR who had the chronic conditions: asthma, diabetes, cancer, heart disease, chronic lung disease, chronic liver disease, or HIV/AIDS (p = 0.62). Rales on clinical examination (p<0.01) and death (p = 0.05) were more common among patients with end point consolidation compared to those with normal CXRs.

**Table 2:** Patient Demographic and Clinical Characteristics by WHO CXR Category.

	Endpoint consolidationn = 385 n(%)	Other infiltrate n = 253 n(%)	Normal n = 79 n(%)	p Value* (Endpoint consolidation vs. Normal
**Demographic Characteristics**				
Median age, years (range)	55 (15–95)	56 (15–96)	45 (15–84)	<0.01
Age category				<0.01
15–39 years	98 (25)	78 (30)	31 (39)	
40–64 years	149 (39)	77 (30)	35 (44)	
≥65 years	138 (36)	98 (39)	13 (16)	
Male Sex	209 (54)	107 (42)	28 (35)	<0.01
“Indigenous ethnicity”	177 (46)	92 (36)	26 (33)	0.06
Ladino ethnicity	193 (50)	153 (60)	51 (65)	0.06
Smoker	51 (14)	24 (10)	4 (5)	0.04
History of Chronic Conditions**	174 (47)	130 (53)	40 (50)	0.62
Asthma / chronic lung disease	60 (16)	55 (22)	20 (25)	0.05
Diabetes	53 (14)	28 (11)	5 (6)	0.06
Heart disease	29 (8)	24 (10)	4 (5)	0.63
Chronic liver disease	11 (3)	6 (2)	1 (1)	0.70
HIV/AIDS	7 (2)	2 (1)	3 (4)	0.39
Income <Q 1,000	246 (84)	170 (85)	51 (86)	0.84
Electricity in the home	348 (94)	227 (91)	72 (92)	0.61
**Clinical Findings**				
Fever (Temperature ≥38°C)	126 (33)	81 (32)	29 (37)	0.49
Fever (by history)	257 (67)	156 (62)	53 (68)	0.88
Hypoxemia ***	130 (40)	85 (40)	23 (34)	0.33
Chills	146 (50)	86 (46)	37 (59)	0.19
Runny nose	183 (49)	140 (57)	42 (53)	0.47
Sneezing	190 (51)	153 (62)	49 (63)	0.06
**Physical Exam Findings**				
Wheezing	90 (25)	78 (32)	28 (36)	0.05
Rales	323 (89)	203 (84)	51 (65)	<0.01
**Outcome**				
Death	47 (13)	17 (7)	4 (5)	0.05

* Fisher’s exact test, except for median age which was performed using Mann-Whitney U test.

** Chronic conditions include asthma, diabetes, cancer, heart disease, chronic lung disease, chronic liver disease, and HIV/AIDS.

*** Oxygen saturation <90% or <88% in Quezaltenango.

Of the 677 patients tested by urine antigen assay, 102 (15%) were positive. Among 252 patients who underwent blood culture, 6 (2%) had *S*. *pneumoniae* isolated (including 2 that also had positive urine antigen testing). Thus, a total of 106 (15%) had ‘pneumococcal infection’. Blood cultures also detected 6 (2%) patients with *S*. *aureus*, 1 with *P*. *aeruginosa*, and 1 with *K*. *pneumoniae*, yielding a total of 114 (16%) with ‘typical bacterial infection’. *M*. *pneumoniae* and/or *C*. *pneumoniae* infections were detected by PCR in 7 patients overall, including 2 patients that also had ‘typical bacterial infection’ (both with a positive *S*. *pneumoniae* urine antigen test) as well as 5 others (3 *M*. *pneumoniae*, 2 *C*. *pneumoniae*). Thus, 119 (17%) patients met the criteria for ‘any bacterial infection.’

Of the 721 included patients, 214 (30%) tested positive for viral infection; 54 (25%) with respiratory syncytial virus, 71 (33%) with influenza, 45 (21%) with parainfluenza, 44 (21%) with adenovirus, and 24 (11%) with human metapneumovirus. Among the 214 patients with a virus detected, multiple viruses were detected in 24 (11%), and ‘any bacterial infection’ was detected in 36 (17%).

Endpoint consolidation was positively associated with ‘pneumococcal infection’, ‘typical bacterial pneumonia’, and ‘any bacterial pneumonia’, compared with normal CXRs (Table 3). All of these associations remained significant after adjusting for age, sex, ethnicity, smoking status, presence of diabetes, and presence of asthma/chronic lung disease. Adjustment changed the diagnostic odds ratio by less than 10% for each of the measured associations. There was no significant association between ‘pneumococcal infection’, ‘typical bacterial infection’, or ‘any bacterial infection’ and the other infiltrate category compared with normal CXRs (Table 4). ‘Viral infection’ was not significantly associated with endpoint consolidation or other infiltrate.

**Table 3:** Association between endpoint consolidation and different types of infections.

Infection Type	Endpoint consolidation N = 385 n(%)	Normal N = 79 n(%)	Unadjusted diagnostic odds ratio (95% CI)	Adjusted diagnostic odds ratio* (95% CI)
**Pneumococcal infection**	72 (19)	6 (8)	3.4 (1.5–10.0)	3.3 (1.3–9.7)
**Typical bacterial infection**	76 (20)	6 (8)	2.9 (1.4–7.9)	2.7 (1.2–7.4)
**Any bacterial infection**	81 (21)	5 (6)	3.2 (1.5–8.6)	2.9 (1.3–7.9)
**Viral infection alone**	106 (28)	23 (29)	0.9 (0.5–1.6)	0.9 (0.5–1.7)

* Adjusted for age, sex, ethnicity, smoking status, presence of diabetes, and presence of asthma/chronic lung disease.

**Table 4:** Association between other infiltrate and different types of infections.

Infection Type	Other Infiltrate N = 253 n(%)	Normal N = 79 n(%)	Unadjusted diagnostic odds ratio (95% CI)	Adjusted diagnostic odds ratio* (95% CI)
**Pneumococcal infection**	32 (13)	6 (8)	1.9 (0.8–5.8)	2.0 (0.8–6.3)
**Typical bacterial infection**	32 (13)	6 (8)	1.8 (0.8–4.8)	1.7 (0.7–4.9)
**Any bacterial infection**	29 (11)	5 (6)	1.7 (0.8–4.8)	1.7 (0.7–4.9)
**Viral infection alone**	84 (33)	23 (29)	1.2 (0.7–2.1)	1.4 (0.8–2.5)

* Adjusted for age, sex, ethnicity, smoking status, presence of diabetes, and presence of asthma/chronic lung disease.

Among 106 patients with ‘pneumococcal infection’, endpoint consolidation was noted in 72, yielding a sensitivity of 68% (95% CI: 59%–76%). Among 611 patients without ‘pneumococcal infection’, the radiograph pattern was not endpoint consolidation (i.e. normal or other infiltrate) for 298, resulting in a specificity of 49% (95% CI: 45%–53%).

## Discussion

Standardized interpretation of adult CXRs identified persons with acute respiratory infection that were more likely to have bacterial infection, independent of age, sex, ethnicity, smoking status, and presence of chronic conditions. ‘Endpoint consolidation’ was associated with bacterial infection, and this was driven by the association with pneumococcal infection, since relatively few infections with other bacterial pathogens were found. Endpoint consolidation was not associated with viral infection alone and other infiltrate was not associated with bacterial or viral infection. Similar to published performance characteristics of pneumococcal urinary antigen testing [31–34], standardized interpretation of adult CXR offers improved sensitivity for pneumococcal infections compared with blood-culture-based definitions and improved specificity compared with clinical definitions.

We also found that endpoint consolidation had moderate sensitivity and specificity for detecting pneumococcal infection, the most common bacterial infection. Because a useful epidemiologic definition of bacterial infections will be both sensitive and specific, we measured the diagnostic odds ratio, which combines measures of sensitivity and specificity into a single indicator, in addition to calculating sensitivity and specificity directly [30]. While neither the sensitivity nor specificity of ‘endpoint consolidation’ was optimal for detecting pneumococcal infection, the balance between sensitivity and specificity for epidemiologic endpoints is a tradeoff. The most sensitive definitions, such as clinical case definitions, are useful for estimating the maximum burden of disease; however, their lack of specificity limits their utility for estimating the impact of specific public health interventions against pneumonia, such as pneumococcal vaccines. Blood-culture-confirmed etiologies, on the other hand, provide a high specificity, but may vastly underestimate the burden of pneumococcal disease and thus the population-level impact of vaccines and other public health interventions against pneumococcal pneumonia [15, 35]. Blood culture, which requires trained personnel, equipment, and specific transport conditions, may not be feasible in many resource-limited settings. The pneumococcal urine antigen test has a sensitivity of 77 to 92% for bacteremic pneumococcal pneumonia and 52 to 78% for non-bacteremic pneumococcal pneumonia; reported specificities have ranged from 67 to 90% [32–35]. This represents higher sensitivity compared with blood culture, and an increased specificity compared with clinical case definitions. However, with a cost of approximately $40 per test, pneumococcal urine antigen is not widely available in resource-poor settings [36]. Standardized interpretation may offer similar performance characteristics while making use of CXRs, which are often obtained for clinical purposes even in resource-limited settings.

Although the utility of standardized interpretation has been previously documented for pediatric CXRs, there is only limited experience with this approach for adult CXRs. Although one study has used standardized interpretation to describe the burden of community-acquired pneumonia in adults, the process was not validated in this analysis [26]. Documentation of an association between ‘endpoint consolidation’ and ‘pneumococcal infection’ in adults suggests that standardized interpretation is useful for measuring pneumococcal infection among adults. Notably, the association between ‘endpoint consolidation’ and ‘pneumococcal infection’ was consistent even after adjusting for age, ethnicity, smoking status, presence of diabetes, and presence of asthma/chronic lung disease. Thus, standardized interpretation is a tool that can potentially be used to measure herd effects in adults resulting from use of pneumococcal conjugate vaccine in children [37, 38]. In recent years, the pneumococcal conjugate vaccine has been increasingly introduced in the routine immunization programs of low- and middle-income countries. However, it is unknown whether the vaccine will result in herd protection in that context. Documentation of herd effects will inform vaccine cost-benefit considerations by giving policy makers a more accurate assessment of the societal benefits of the vaccine program.

This analysis has limitations. CXRs and blood cultures were obtained at physician discretion and were not performed for all patients. We are uncertain of the impact that exclusion of those patients may have had on our results. Patients for whom CXRs were obtained were more likely to have rales, a clinical finding associated with bacterial pneumonia, on physical exam; therefore, our hypothesis is that patients in the cohort without CXRs were less likely to have bacterial pneumonia and more likely to have viral pneumonia compared with those patients for whom CXR was obtained. Because CXR findings among patients with viral infections were relatively equally distributed between the three CXR classifications, our hypothesis is that addition of these patients would have little effect on the associations we observed. Secondly, patients who did not have clinical pneumonia may have been included since the criteria for acute respiratory infection were quite broad, likely including patients with upper respiratory infections who did not require that the patient have suggestive clinical features for pneumonia. The majority of these patients would likely have normal CXR findings and negative testing for bacterial pathogens, resulting in a minimal impact on the observed association between bacterial pneumonia and ‘endpoint consolidation.’ Thirdly, multiple study physicians examined patients; therefore, there may have been some inter-observer variability in the physical exam findings. However, since the radiologists did not have access to the results of the physical exam findings when making interpretations, this variability did not affect the findings. Additionally, since the diagnostic gold standards for bacterial pneumonia, urine antigen and blood culture, have limited sensitivity, some patients may have been misclassified. Since we had no assay to test for non-pneumococcal, non-bacteremic pneumonia, very few non-bacteremia pneumonia cases with etiologies other than *S*. *pneumoniae* were found. Perhaps this is the reason that a bacterial or viral etiology was identified for only 45% of patients with ‘endpoint consolidation’ and 48% of patients with ‘other infiltrate.’ If, as we suspect, more sensitive diagnostics would identify more patients with bacterial infections among those with ‘endpoint consolidation’, the strength of the observed association between ‘endpoint consolidation’ and ‘any bacterial infection’ would increase. Finally, our conclusions about associations between pneumococcal infections and standardized interpretation findings cannot be generalized to pneumonia caused by other pathogens or to settings where the distribution of pathogens may be different from Guatemala. However, given that prospective surveillance identified *S*. *pneumoniae* as the most commonly identified bacterial pathogen in Guatemala, consistent both with our findings and data in other countries, our findings may be generalizable to a variety of settings [2, 28]. Finally, PCR of NP/OP swabs may result in detection of infections in healthy persons; therefore there may have been some misclassification among those considered to have viral infections and may explain the relatively high proportion of persons with normal CXRs and ‘viral infection’ [39].

By identifying adults more likely to have pneumococcal infections, standardized interpretation of adult CXRs may be useful for measuring the indirect effects of vaccinating infants with the pneumococcal conjugate vaccine. Although further work will be necessary to validate and optimize the use of standardized interpretation in adults, this use of CXRs, which are widely available, even in resource-poor settings, is a feasible way to estimate adult pneumonia disease burden and the impact of public health interventions such as pneumococcal conjugate vaccine on adult pneumonia. As Guatemala introduced the 13-valent pneumococcal conjugate vaccine in November 2012, standardized interpretation of adult CXR may be useful for measuring indirect effects in this setting.
